# Diet Reconstruction Under Limited Prior Information: Dietary Contributions and Isotopic Niche of *Metridium senile* in the North Yellow Sea

**DOI:** 10.3390/biology14111508

**Published:** 2025-10-28

**Authors:** Yongsong Zhao, Xiujuan Shan, Guangliang Teng, Shiqi Song, Yunlong Chen, Xianshi Jin

**Affiliations:** 1 State Key Laboratory of Mariculture Biobreeding and Sustainable Goods, Key Laboratory of Sustainable Development of Marine Fisheries, Ministry of Agriculture and Rural Affairs, Yellow Sea Fisheries Research Institute, Chinese Academy of Fishery Sciences, Qingdao 266071, Chinatenggl@ysfri.ac.cn (G.T.);; 2 Laboratory for Marine Fisheries Science and Food Production Processes, Qingdao Marine Science and Technology Center, Qingdao 266237, China; 3 Shandong Changdao National Observation and Research Station for Fisheries Resources, Yantai 265800, China; 4 College of Marine Living Resource Sciences and Management, Shanghai Ocean University, Shanghai 201306, China

**Keywords:** stable isotopes, Bayesian mixing model, trophic position, isotopic niche, limited prior dietary information, *Metridium senile*

## Abstract

Biomass of the plumose anemone *Metridium senile* has surged in the benthic ecosystem of the North Yellow Sea in recent years. Understanding its diet and the proportional contributions of food sources is essential for assessing the ecological consequences of this expansion. The species is often characterized as a passive suspension feeder, yet laboratory feeding trials have documented shrimp consumption. Because prior dietary information from the region is scarce, conventional stable isotope approaches are poorly constrained. We developed an integrative framework coupling trophic position estimation, isotopic niche metrics, spatial point pattern analysis, and a Bayesian mixing model to improve diet attribution under limited prior information and to test whether *M. senile* preys on small-bodied and juvenile teleosts and invertebrates under natural conditions. Our analyses showed that: (i) *M. senile* occupied a high trophic position (TP = 3.09 ± 0.25), exceeding those estimated for putative predators in our dataset, implying weak top-down control; (ii) in isotopic niche analyses, *M. senile* showed high posterior probabilities of occurring within the niches of cephalopods and medium-sized fishes (78.30% and 63.04%, respectively), consistent with shared prey and inconsistent with a strictly suspension-feeding strategy; (iii) mixing space diagnostics informed by spatial point pattern analysis indicated that including small-sized fishes and shrimps as sources was necessary to reconcile the elevated TP; and (iv) the Bayesian mixing model estimated that small-bodied and juvenile teleosts and invertebrates supplied most long-term nutrition (posterior mean ≈ 0.65), with the remainder from suspension-derived sources, consistent with an opportunistic generalist rather than a strict suspension feeder. Sustained predation on small-bodied and juvenile teleosts and invertebrates could suppress early fish recruitment, impose top-down control on forage species, and alter the local food web structure. Management should monitor *M. senile* (size structure, population density, and co-occurrence with juveniles and forage biota) and consider targeted removals and seafloor litter cleanups in priority habitats. The framework is applicable to diet studies with limited prior information; adding δ^34^S, compound-specific amino-acid isotopes (CSIA-AA), and DNA-based dietary evidence should further sharpen source discrimination.

## Simple Summary

The sea anemone (*Metridium senile*) has become much more common on the seafloor of the North Yellow Sea, but its true diet in nature has been unclear. We asked two questions: what role does this anemone play in the local food web, and how can we trace its diet when background information is limited? We compared the stable isotopes in anemone tissues with those of likely foods and used a clear set of tests to track where its nutrition comes from. The results show that this anemone is not just a passive filter of tiny particles. It feeds heavily on small fishes and shrimps, as well as other small seafloor animals, and sits high in the food chain—higher than some species usually thought of as predators. About two thirds of its long-term diet appears to come from small fishes and invertebrates, with the rest from suspended material. Ongoing feeding on young fishes could reduce the number that survive to adulthood and reshape local ecosystems. We suggest monitoring its size, density, and overlap with young fishes and piloting targeted removals with seafloor litter clean-ups in priority areas. Our framework can be applied to diet studies when food-source data are limited.

## 1. Introduction

Sea anemones (*Actiniaria*) are highly adaptable, with approximately 1200 species distributed worldwide across all latitudes and depth ranges [1]. In China, they occur mainly along the coasts, typically attached to rock surfaces or inhabiting mixed sandy and muddy substrates. Species richness shows a clear geographic gradient: highest in the South China Sea, intermediate in the Yellow and Bohai Seas, and lowest in the East China Sea, with substrate type, water depth, and salinity as key determinants of their distribution [2]. Recent studies indicate that *Metridium senile* (Linnaeus, 1761) has become a dominant component of benthic communities in the North Yellow Sea, with biomass increasing sharply in recent years and, in some areas, dominance exceeding that of crustaceans and mollusks [2,3]. The North Yellow Sea is a semi-enclosed marine ecosystem bounded by the Miaodao Archipelago, the Shandong and Liaodong Peninsulas, and the Korean Peninsula. Its coasts support major mariculture and fishing grounds and are strongly affected by human activities and environmental change. It also functions as a key migratory corridor and as spawning and foraging habitats for many fisheries species in the Yellow and Bohai Seas. The Yellow Sea Ecoregion (YSE), identified by the Worldwide Fund for Nature (WWF) as one of 43 globally prioritized marine ecoregions and the only one that includes Chinese waters, has outstanding ecological value and high conservation significance.

**Figure 1:**
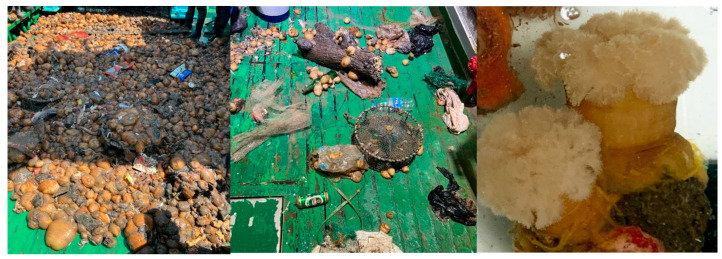
Records of the sea anemone *M. senile* from bottom trawl surveys in the North Yellow Sea.

Suitable attachment substrates likely facilitated the rapid increase in *M. senile* in the North Yellow Sea [2,3]. Previous work found that the accumulation of anthropogenic seabed macro-debris (ASMD) is an important predictor of *M. senile* distribution in the region, with a significant positive association between the two in spatial analyses [3]. Because the species requires firm surfaces for attachment, the widespread presence of ASMD has provided functional habitat for growth and reproduction and mobile vectors for regional spread, supplying both habitat conditions and additional dispersal nodes that have helped drive the outbreak. Recent bottom trawl surveys in the North Yellow Sea have documented *M. senile* attached to large items of ASMD, including fishing nets, glass bottles, rubber gloves, textiles, and plastics (Figure 1).

Beyond suitable attachment substrates, ample food supply may also be an external driver of the rapid population increase in *M. senile* in the North Yellow Sea. Existing studies generally characterize *M. senile* as a passive suspension feeder that derives energy primarily from zooplankton [4,5,6]. A small number of studies also indicate that *M. senile* preys on small-bodied and juvenile teleosts and invertebrates, with shrimp consumption observed under laboratory conditions [7,8,9]. However, the dietary ecology of this species in Chinese coastal waters remains poorly documented. Importantly, passive suspension feeding and predation on small-bodied and juvenile teleosts and invertebrates imply different food web pathways and distinct potential impacts on benthic communities and ecosystem structure in the North Yellow Sea. Accurate identification of diet composition and the relative contributions of sources is therefore essential to understanding the ecological consequences of its population outbreak.

In recent years, rapid advances in stable isotope analysis have provided an important way to quantify dietary source contributions in animal dietary ecology [10]. The carbon and nitrogen stable isotope composition of consumer tissues integrates the isotopic signatures of assimilated foods over time and is therefore closely linked to dietary sources. Carbon isotopes (δ^13^C) are commonly used to constrain the primary source spectrum, whereas nitrogen isotopes (δ^15^N), which exhibit predictable enrichment across trophic levels, are widely used to estimate consumer trophic position [11,12,13,14]. However, consumer diets typically involve multiple sources, and this complexity and associated uncertainty make it difficult to apportion contributions from carbon and nitrogen isotopes alone. To address this challenge, Bayesian stable isotope mixing models have been developed and are now widely used in studies of aquatic food webs [15]. These models use two or more isotopes to estimate the relative nutritional contributions of candidate sources to a consumer, and they can incorporate environmental and biological covariates to reduce uncertainty and improve the fidelity of the estimates. Although stable isotope analysis is a key tool for tracing consumer nutrition, it has clear limits when prior information on dietary sources is scarce. The isotopic composition of consumer tissues represents an amalgamation of assimilated materials from multiple sources over time, and therefore cannot precisely delineate specific dietary origins. For instance, a consumer δ^13^C value of −21‰ could result from the proportional assimilation of two distinct sources with values of −24‰ and −18‰, or exclusively from a single source at −21‰ plus the relevant trophic discrimination factor (Δ^13^C). This mathematical multiplicity of solutions necessitates the application of ecological priors to constrain biologically plausible interpretations. In the mixing space, many combinations are mathematically feasible. Without ecological constraints, Bayesian mixing models may return biologically implausible solutions and lead to misinterpretation. These models also apportion only relative contributions among the specified sources, so they are sensitive to source omission or misclassification. Therefore, background information on candidate food sources should be assembled and used before modeling to constrain the mixing space and reduce uncertainty.

However, for many marine organisms, traditional approaches provide insufficient prior information (for example, in invertebrates for which stomach content analysis is infeasible), which represents a challenge for the application of Bayesian stable isotope mixing models to quantify the dietary sources of specific marine consumers. The focal species in this study, *M. senile*, exemplifies this constraint. Sea anemones are cnidarians with a single orifice that serves both ingestion and egestion; internally they possess a gastrovascular cavity rather than a complete gastrointestinal tract [16]. The cavity contains digestive fluids and cells, and prey captured by nematocysts is rapidly immobilized and broken down, often beyond recognition, so conventional stomach-content analysis rarely yields reliable dietary information. In addition, the cavity configuration is highly susceptible to contamination during molecular diet analyses, and the paucity of data on this species’ diet in the North Yellow Sea further complicates indirect inference of dietary sources. Consequently, limited background information on candidate sources poses a major challenge to precise source attribution for *M. senile* in the region. Although our previous work used stable isotopes to explore the feeding ecology of North Yellow Sea anemones, it did not focus on identifying dietary sources as the central question [8].

To address these limitations, we developed an approach for dietary source attribution under limited prior information. The approach integrates trophic position analysis, isotopic niche comparison, and spatial point-pattern analysis in isotopic space to qualitatively identify candidate dietary source categories for *M. senile* in the North Yellow Sea. We then applied a Bayesian stable isotope mixing model (SIMMR) to quantify the proportional contributions of each source and to characterize their posterior distributions.

Based on the above methodological design, this study pursued two objectives: (1) to evaluate under field conditions whether *M. senile* in the North Yellow Sea consumes small-bodied and juvenile teleosts and invertebrates and to quantify the contributions of dietary sources; and (2) to develop and test stable isotope approaches for dietary source attribution when prior information on sources is limited. The findings provide methodological guidance for dietary source attribution of marine consumers under limited prior information and a scientific basis for understanding the potential cascading and broader ecological effects of *M. senile* outbreaks on demersal fisheries species in the North Yellow Sea in environments influenced by anthropogenic seabed macro-debris.

## 2. Methods

### 2.1. Study Area and Sample Collection

**Figure 2:**
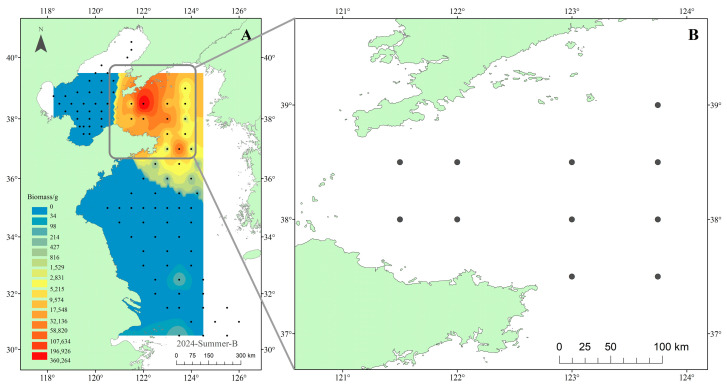
Map showing the sampling sites for (**A**) the biomass survey of *M. senile* in the Yellow and Bohai Seas (August 2024), where the distribution was interpolated using Inverse Distance Weighting (IDW), and (**B**) the stable isotope sampling in the North Yellow Sea (May 2021).

Stable isotope samples were collected in May 2021 during the Yellow Sea Fisheries Resources and Environment Survey in the North Yellow Sea (121.5°–123.75° E, 37.5°–39.0° N). Eleven stations were sampled from the research vessel Zhongyu Ke 102 (Figure 2B). For context, we also used the summer 2024 fisheries survey across the Yellow and Bohai Seas (95 stations) conducted by the research vessel Beidou and Zhongyu Ke 102 to map the distribution of *M. senile* biomass by inverse distance weighting (IDW). The survey network and the IDW-interpolated biomass surface are shown in Figure 2A and indicate that the North Yellow Sea is the main high-biomass area for this species. Both surveys used single vessel bottom trawling; the trawl net was 44.9 m in total length with a mouth height of 8 m and width of 12 m, headrope and footrope lengths of 60 m each, a body mesh of 200 mm (400 meshes), and a codend mesh of 38 mm (831 meshes). Each tow lasted 1 h at a speed of 3 kn.

After collection, the catch samples were identified to species and subjected to biological measurements onboard, followed by freezing for preservation. These samples were then transported to the laboratory for stable isotope analysis. Surface sediment samples were collected using a grab sampler (5 L: 305 mm × 150 mm × 480 mm), with sedimentary organic matter (SOM) samples taken from approximately 1 cm depth of the sediment surface [17]. A 2.5 L bottom water sample was collected and prefiltered through a 200-μm nylon mesh to remove large inorganic particles and plankton. The resulting filtrate was then vacuum filtered through a precombusted (4 h at 450 °C) Whatman GF/F glass fiber filter to obtain suspended particulate organic matter (POM) samples. Phytoplankton and zooplankton were collected and processed as distinct functional groups, as they are commonly consumed by predators at higher trophic levels through non-selective feeding. Phytoplankton samples were collected at each station using vertical hauls from bottom to surface with a Type III plankton net (mouth area: 0.1 m^2^, mesh size: 77 μm), with the collected material transferred to 1 L polyethylene bottles. Zooplankton were sampled with a Type II plankton net (mouth area: 0.08 m^2^, mesh size: 160 μm) through vertical tows from bottom to surface waters. To minimize isotopic contamination from gut contents, zooplankton samples were maintained in pre-filtered seawater for 2 h to allow for complete gut evacuation before further processing. All plankton samples were filtered onto pre-combusted (450 °C for 4 h) Whatman GF/F glass fiber filters (nominal pore size 0.7 μm) using a vacuum filtration system, following established methods [18].

Individuals were identified to the species level based on external morphological characteristics (e.g., body shape, fin structure, and coloration), using the taxonomic keys provided in Fishes of the Bohai Sea and Yellow Sea [19], Atlas of Benthonic Animals of the Yellow Sea and Bohai Sea [20], and Mollusks of the Yellow Sea and Bohai Sea [21]. All species were cross-checked on FishBase (https://www.fishbase.se) (accessed on 12 October 2024), SeaLifeBase (https://www.sealifebase.org) (accessed on 12 October 2024), and the World Register of Marine Species (WoRMS; https://www.marinespecies.org) (accessed on 12 October 2024), with their Latin and scientific names standardized. At each sampling station, we collected not only individuals of *M. senile* but also virtually the entire demersal catch for the purpose of comprehensive trophic reconstruction and baseline establishment. A total of 238 samples representing 61 species were analyzed, with four parameters measured for each sample (δ^13^C, δ^15^N, %C, and %N), to establish the baseline for stable isotope analysis and to delineate the isotopic niche.

### 2.2. Functional Groups

Food webs are highly complex, and their full reconstruction is often constrained by limited data availability and computational feasibility. To elucidate dominant energy flows and key trophic levels while reducing parameter uncertainty, researchers commonly employ a simplified food web approach. This involves aggregating species with similar ecological roles into functional groups, retaining major trophic pathways, and omitting weak interactions, thereby enhancing model interpretability and applicability [22]. For the demersal ecosystem of the North Yellow Sea, we defined 16 functional groups for stable isotope analysis. These included three basal resource groups (phytoplankton, POM, and SOM) and 13 consumer groups, which collectively represent the trophic structure of the system (Table 1). As most fish in the study area are benthic omnivores, diet alone proved insufficient for distinguishing functional groups. Since body size reflects gape limitation and predatory capacity, and fish of comparable size and feeding ability often utilize similar resources, we categorized fish into two groups based on average adult body length: small-sized fishes (<15 cm) and medium-sized fishes (15 to 30 cm). Due to their distinct ecological role, jellyfishes were separated from plankton and treated as an independent group. This grouping scheme effectively represents the trophic organization of the demersal ecosystem in the study area (Table 1).

### 2.3. Stable Isotope Analysis and Models

Tissue samples for stable isotope analysis were collected according to different biological categories. For sea anemones, the foot disk was collected [8]. For fish, white muscle tissue near the first dorsal fin was collected, with the skin, bones, and blood removed. For shrimp, abdominal muscle tissue was collected [23]. For crabs, muscle from the first cheliped was collected for larger individuals, while abdominal muscle was collected for smaller crabs. For cephalopods, mantle and arm muscle tissues were collected. For gastropods, the shell was removed and the muscle tissue was collected. For bivalves, the adductor muscle was collected. For other small invertebrates, if sufficient white muscle tissue was not available, the entire individual was used for stable isotope analysis. Before δ^13^C analysis of SOM samples and whole small crustaceans, the samples were treated to remove the influence of inorganic carbon [13]. The method involved dividing these isotope samples into two portions, with one half treated with acid (1 mol/L hydrochloric acid) to remove inorganic carbon for δ^13^C analysis, and the other half not directly acidified for δ^15^N analysis. After preparation in the laboratory, all samples were dried at 60 °C for 48 h until a constant weight was achieved. The dried samples were then ground using a ball mill, placed into tin capsules, and subjected to stable isotope analysis. Glass fiber filter samples were scraped from the filters, placed into tin capsules, and analyzed for stable isotopes [24].

Δ*R* = [(*X*_sample_ − *X*_standard_)/*X*_standard_] × 10^3^(‰)

where *R* represents the ^13^C or ^15^N, and X represents the ratio of ^13^C/^12^C or ^15^N/^14^N. The precision of the measured stable carbon and nitrogen isotope ratios met the requirements of δ^13^C ≤ ±0.2‰ and δ^15^N ≤ ±0.3‰.

The trophic discrimination factors (TDFs) used in this study were set as Δ^13^C = 1.0 ± 0.2‰ and Δ^15^N = 3.4 ± 0.2‰ (mean ± SD), following the empirical recommendations for aquatic food webs by Post (2002) [13]. Data with C/N ratios greater than 3.5 were excluded as much as possible to minimize the interference from lipids.

To quantify niche overlap between functional groups, we used the R package nicheROVER (version 1.1.2) to estimate probabilistic niche regions in isotopic space (δ^15^N, δ^13^C) via posterior sampling from a multivariate normal model with an inverse-Wishart prior, and we computed niche size and pairwise directional overlap at α = 0.95 [25]. For settings with limited information on dietary sources, we combined trophic position analysis, isotopic niche overlap, and spatial point pattern analysis to identify candidate sources for *M. senile* in the North Yellow Sea. We then applied the Bayesian stable isotope mixing model SIMMR to estimate posterior distributions of source contributions, thereby improving attribution accuracy under limited prior information. The isotopic mixing space is the region in the two-dimensional δ^13^C–δ^15^N plane bounded by the convex hull (mixing polygon) formed by the candidate source end-members [26,27]. A consumer’s isotopic values must fall within this polygon for those sources to be able to mix to produce the observed signature. Accordingly, before fitting the Bayesian mixing model, we verified that the majority of consumer isotope values fell within the mixing polygon defined by the candidate sources. We quantified the distribution of isotopic data in mixing space using spatial point pattern analysis with the R package splancs (version 2.01-45). At each of 1500 iterations, we drew source means and standard deviations, together with trophic enrichment factors (TEFs), from their specified uncertainty distributions. We applied the TEFs to adjust source values, constructed the convex hull of the corrected sources, and recorded whether each consumer observation fell inside the hull. On a regular grid, we then computed the proportion of iterations in which each cell lay inside the hull and visualized these coverage frequencies as heat maps and contour plots. This approach addresses two questions: (1) whether the observed consumer isotope values can be explained by the specified sources, that is, whether consumer points fall within the mixing polygon; and (2) how the feasible mixing region is distributed probabilistically when uncertainty in sources and TEFs is propagated. Systematic placement of consumer observations outside the hull may indicate measurement error, omission of an important source, or misspecified TEFs [26,27,28]. We fitted Bayesian stable isotope mixing models using the R package SIMMR (version 0.5.1.217). As the successor to SIAR, SIMMR retains the core functionality of its predecessor while providing more flexible model structures, a streamlined workflow, and improved visualization tools [29].

### 2.4. Trophic Position Estimation

Estimating consumer trophic position from δ^15^N requires the selection of one or more baseline taxa to serve as an isotopic reference (hereafter, baselines). The appropriate choice of these baselines is critical for stable isotope studies in marine ecosystems [13]. Baselines can vary substantially across different marine ecosystems—and even among guilds within the same system—due to differences in the sources of matter and energy [30]. In complex food webs characterized by multiple energy pathways, multiple baselines are often necessary to adequately represent the trophic structure [31]. Consequently, accurate baseline selection is a fundamental prerequisite for obtaining reliable trophic-position estimates.

In studies of the Yellow Sea ecosystem, zooplankton or benthic gastropods and bivalves are often used as a single baseline to represent trophic level 2. However, reliance on these potential baselines can differ markedly among consumers, so using only one baseline may bias trophic position estimates. To identify suitable baselines for major demersal consumers in the North Yellow Sea, we applied the two-source mixing model of Post (2002) [13] to quantify the relative contributions of zooplankton versus benthic gastropods and bivalves for each consumer, and then calculated trophic position using the resulting baseline assignments. The model is:

*α*_base 1_ = (*δ*^13^C_consumer_ − *δ*^13^C_base 2_)/(*δ*^13^C_base 1_ − *δ*^13^C_base 2_)

where α_base 1_ denotes the relative contribution of baseline 1 to the consumer; δ^13^C_consumer_ is the consumer’s carbon isotope value; δ^13^C_base 1_ and δ^13^C_base 2_ are the carbon isotope values of baselines 1 and 2, respectively.

Trophic position was calculated following Post (2002) [13]:

*TP* = 2 + [*δ*^15^N_consumer_ – (*δ*^15^N_base 1_ × *α* + *δ*^15^N_base 2_ × *α*_base 2_)]/3.4

*α*_base 2_ = 1 − *α*_base 1_

where *TP* is trophic position; δ^15^N_consumer_ is the consumer’s nitrogen stable isotope ratio; δ^15^N_base 1_ and δ^15^N_base 2_ are the nitrogen stable isotope ratios of baselines 1 and 2; α_base 1_ and α_base 2_ are the relative contributions of baselines 1 and 2 to the consumer.

Zooplankton and benthic gastropods and bivalves were used as baseline taxa (base 1 and base 2, respectively). The baseline trophic level was set to 2, and the trophic enrichment in δ^15^N (Δ^15^N) was 3.4‰.

### 2.5. Data Analysis

Maps of sampling stations were produced in ArcGIS (version 10.2; Esri, Redlands, CA, USA). Mixing-model, niche, and point-pattern analyses were conducted in R 4.5.0 (R Foundation for Statistical Computing, Vienna, Austria) using the packages SIMMR (version 0.5.1.217), nicheROVER, (version 1.1.2) and splancs. (version 2.01-45) Statistical analyses and data visualization were performed in R 4.4.1, Microsoft Excel, and Adobe Illustrator 2023.

## 3. Results

### 3.1. Stable Isotope Signatures of Demersal Fisheries Species in the North Yellow Sea

**Figure 3:**
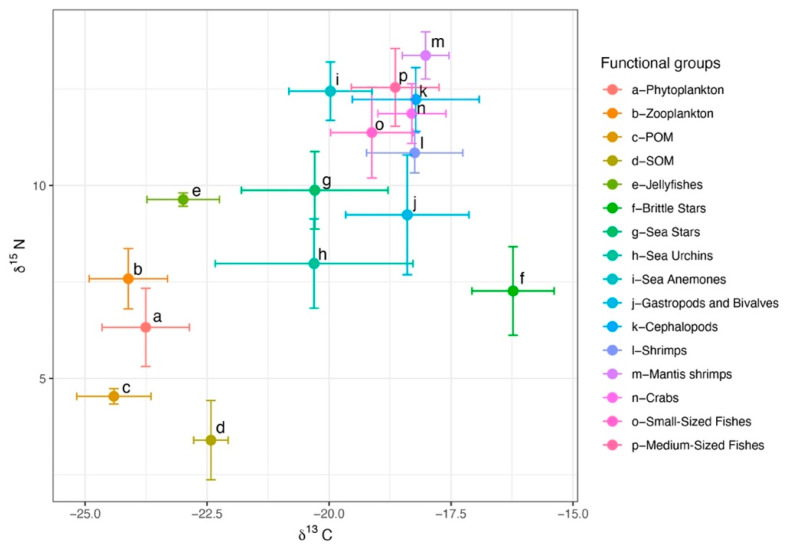
Stable isotope distributions of major fisheries species in the North Yellow Sea.

A total of 238 samples from the North Yellow Sea were analyzed for δ^13^C and δ^15^N. For consumers, δ^13^C ranged from −25.15 to −15.40‰ (mean ± SD = −18.88 ± 1.52‰) and δ^15^N from 5.77 to 14.27‰ (11.50 ± 1.76‰). Basal producers (phytoplankton, POM, SOM) had δ^13^C of −25.40 to −21.88‰ (−23.22 ± 1.04‰) and δ^15^N of 1.53 to 7.33‰ (4.50 ± 1.57‰). The broad δ^13^C spread among consumers indicates diverse carbon sources. Among consumer groups, sea urchins showed the greatest isotopic niche width as measured by the carbon range (CR), whereas brittle stars had the largest nitrogen range (NR). Sea anemones occupied a relatively high trophic position and, in isotopic space, clustered with cephalopods, fishes, shrimps, and crabs. Across groups, δ^15^N increased in the order fishes > invertebrates > zooplankton > phytoplankton > POM > SOM. Basal producers clustered in the lower left of isotopic space, and consumer groups shifted toward the upper right with increasing trophic position (Figure 3).

### 3.2. Trophic Position Analysis

**Figure 4:**
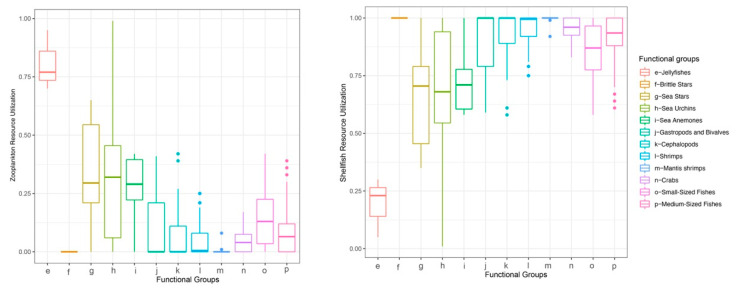
Consumer reliance on baseline taxa by functional group.

**Figure 5:**
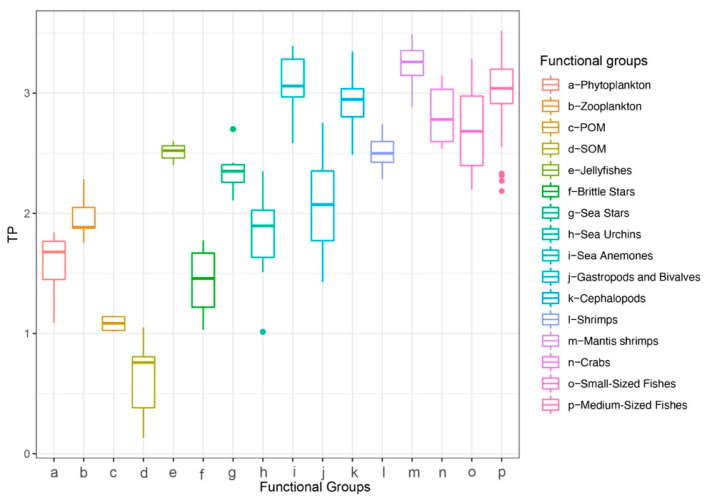
Trophic position (TP) by functional group in the North Yellow Sea.

Baseline assignment indicated that the median reliance of most consumers on gastropods and bivalves exceeded 50%, except for jellyfishes, which relied more heavily on zooplankton (Figure 4). Therefore, for subsequent trophic-position calculations, the zooplankton baseline was applied to jellyfishes, and the gastropod and bivalve baseline was used for all other consumers. The estimated trophic positions (TP) of major groups ranged from 0.13 to 3.52. The sea anemone exhibited a high TP (mean ± SD = 3.09 ± 0.25), which was comparable to those of cephalopods (2.93 ± 0.24), mantis shrimps (3.23 ± 0.18), and medium-sized fishes (3.02 ± 0.27) (Figure 5). Using standard trophic level (TL) thresholds (TL 1: 0.5 < TP ≤ 1.5; TL 2: 1.5 < TP ≤ 2.5; TL 3: 2.5 < TP ≤ 3.5; TL 4: 3.5 < TP ≤ 4.5), we categorized the groups as follows: phytoplankton, POM, and SOM occupied TL 1; zooplankton, gastropods and bivalves, brittle stars, sea urchins, and sea stars were assigned to TL 2; and sea anemones, jellyfishes, cephalopods, shrimps, crabs, mantis shrimps, small-sized fishes, and medium-sized fishes fell within TL 3 (Table 2). In summary, *M. senile* occupies a relatively high trophic position within the studied ecosystem, comparable to that of other typical predatory groups.

### 3.3. Isotopic Niche Overlap Analysis

**Figure 6:**
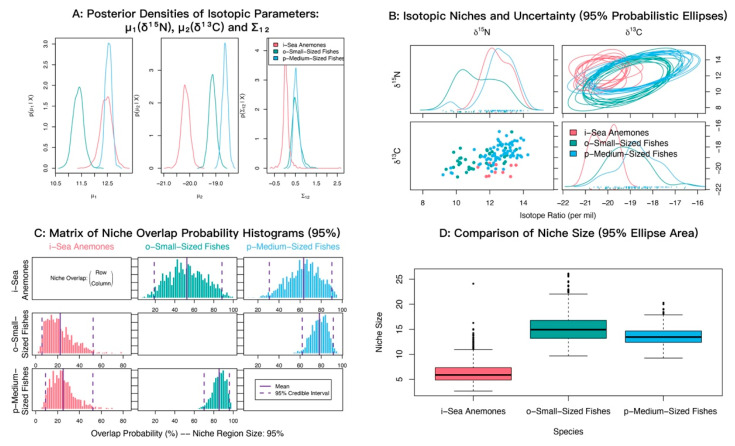
Isotopic niche overlap between the sea anemone *M. senile* and major fish groups in the North Yellow Sea, estimated using the probabilistic Bayesian framework implemented in NicheROVER. (**A**) Posterior densities of the core isotopic parameters (μ_1_: δ^15^N, μ_2_: δ^13^C) and their covariance (Σ_12_). (**B**) Biplot of δ^13^C vs. δ^15^N showing raw data points and ten posterior realizations of the 95% probabilistic niche ellipses. (**C**) Posterior distributions of pairwise directional niche overlap; solid and dashed vertical lines indicate the posterior mean and 95% credible interval, respectively. Overlap is defined as the probability that an individual from a row group falls within the 95% niche region of a column group. (**D**) Posterior distributions of the 95% niche area for each group, shown as boxplots. All axes are in units of per mil (‰).

In δ^13^C–δ^15^N space, sea anemones and medium-sized fishes showed strongly overlapping posterior distributions of δ^15^N with similar means (12.45‰ and 12.54‰), both higher than small-sized fishes (11.37‰). For δ^13^C, medium-sized fishes were most enriched (−18.64‰), sea anemones were most depleted (−20.16‰), and small-sized fishes were intermediate (−19.12‰) (Figure 6A). Isotopic niche analyses indicated appreciable overlap between sea anemones and both fish groups (Figure 6B). Posterior niche-overlap probabilities (NicheROVER) were high for medium-sized fishes occurring within the niche of small-sized fishes (85.81%, 95% credible interval: 72 to 96%) (Table 3). The probabilities that sea anemones fall within the niches of medium-sized fishes and small-sized fishes were 63.04% and 52.68%, respectively (Figure 6C; Table 3).

**Figure 7:**
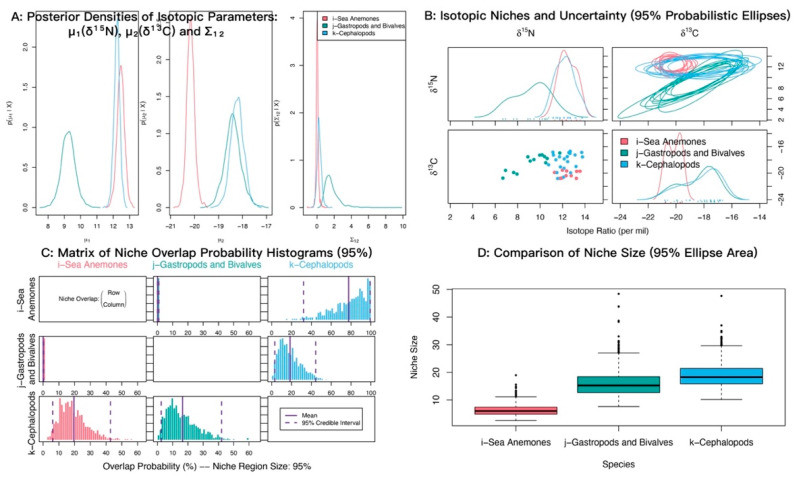
Isotopic niche overlap between sea anemones (*M. senile*) and major mollusk groups (gastropods and bivalves; cephalopods) in the North Yellow Sea (NicheROVER).

In δ^13^C–δ^15^N isotopic space, sea anemones and cephalopods showed strongly overlapping posterior distributions of δ^15^N with similar means (12.45‰ and 12.23‰), both higher than gastropods and bivalves (9.24‰). For δ^13^C, cephalopods and gastropods and bivalves also overlapped closely with similar means (−18.22‰ and −18.39‰), whereas sea anemones were most depleted (−20.16‰) (Figure 7A). Isotopic niche analyses indicated substantial overlap between sea anemones and cephalopods (Figure 7B). The posterior probability that sea anemones fall within the cephalopod niche was 78.30% (95% credible interval 34–100%) (Figure 7C; Table 4).

**Figure 8:**
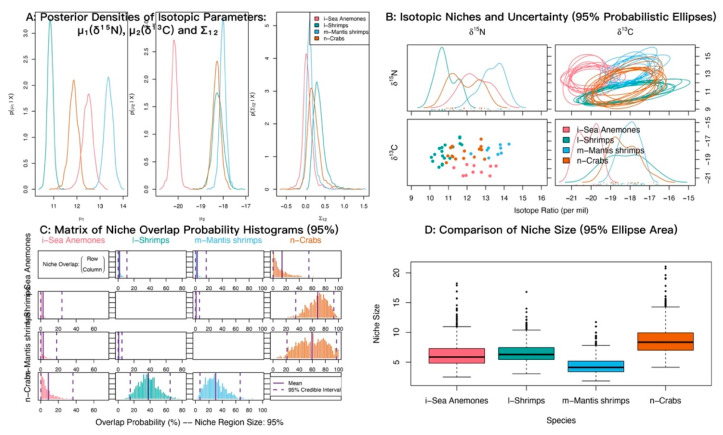
Isotopic niche overlap between sea anemones (*M. senile*) and shrimps, crabs, and mantis shrimps in the North Yellow Sea (NicheROVER).

At α = 0.95, the posterior probabilities that sea anemones fall within the niches of shrimps, crabs, and mantis shrimps were low (Figure 8; Table 5). The probabilities that shrimps and mantis shrimps fall within the crab niche were 68.22% and 53.37%, respectively.

**Figure 9:**
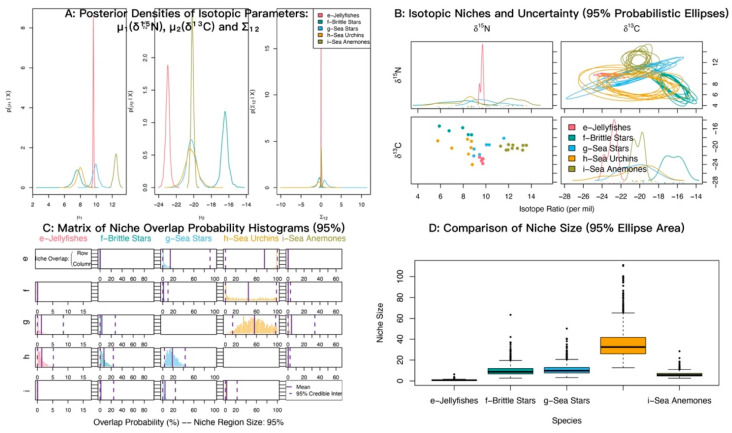
Isotopic niche overlap between sea anemones (*M. senile*) and jellyfishes, brittle stars, sea stars, and sea urchins in the North Yellow Sea (NicheROVER).

At α = 0.95, the posterior probabilities that sea anemones (*M. senile*) fall within the niches of jellyfishes, brittle stars, sea urchins, and sea stars were low (Figure 9; Table 6). By contrast, the probabilities that jellyfishes, brittle stars, and sea stars fall within the sea urchin niche were relatively high.

Isotopic niche overlap analysis indicated that sea anemones (*M. senile*) had high posterior probabilities of occurring within the niches of cephalopods, medium-sized fishes, and small-sized fishes (78.30%, 63.04%, and 52.68%, respectively). This pattern suggests shared prey and is inconsistent with a purely suspension-feeding strategy. Because *M. senile* is sessile, attaches to rocks and anthropogenic seabed macro-debris, and captures prey by contact with its tentacles rather than active pursuit, we infer opportunistic carnivory, similar to that of cephalopods. Within colonized areas, accessible prey within its ingestible size range may be taken opportunistically.

### 3.4. Spatial Point Pattern Analysis (Mixing-Space Diagnostics)

Guided by the trophic position and niche-overlap results, we infer that, in addition to passive suspension feeding, sea anemones (*M. senile*) likely prey on small fisheries species (high TP and proximity in isotopic space to cephalopods and small- to medium-sized fishes). On this basis, we specified source groups for mixing-space diagnostics and subsequent modeling as follows: suspension-derived sources were zooplankton, POM, and SOM; fishery-organism sources were small-sized fishes and shrimps. Adult gastropods and bivalves were considered not ingestible for *M. senile* because shell structure and locomotion make capture by tentacles unlikely; if larvae or spat are taken, they belong with zooplankton. Adult crabs were likewise treated as not ingestible and unlikely to be subdued. This source selection reflects the gape limits and contact capture feeding mode of sea anemones.

**Figure 10:**
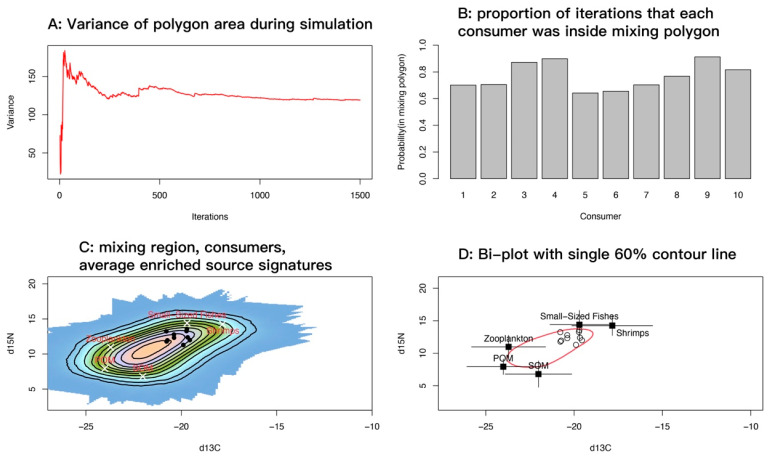
Diagnostic plots for the spatial point pattern analysis of the mixing space. (**A**) Running variance of mixing-polygon area across iterations; the curve leveling off indicates that the number of iterations is sufficient. (**B**) For each consumer, the proportion of iterations in which it lay inside the mixing polygon; values closer to 1 indicate that the specified sources consistently explain the isotopic signature, whereas values < 0.2 suggest frequent exclusion and warrant checking TEFs, source categories, or data quality. (**C**) Feasible mixing region shown as grid-cell coverage frequencies (probability surface) under uncertainty, overlaid with TEF-corrected source means. (**D**) Bivariate scatterplot with a single red 60% contour.

Accounting for uncertainty in trophic enrichment factors (TEFs), we implemented 1500 Monte Carlo iterations in R, using splancs for convex-hull and geometric operations. At each iteration, the model sampled TEFs and source means and standard deviations from their uncertainty distributions, adjusted source values accordingly, constructed the convex hull of the corrected sources, and recorded whether each consumer observation fell inside the hull. On a regular grid, coverage frequency across iterations summarized the feasible mixing region and was visualized as heat maps and contour plots. The running variance of hull area declined rapidly and stabilized after a few hundred iterations, indicating that the iteration count was sufficient for stability and convergence (Figure 10A). For each consumer, the model computed the proportion of iterations in which it lay inside the mixing polygon, as a measure of the stability with which the specified sources explain its isotopic signature. Most samples showed high proportions (>0.7), and several approached or exceeded 0.9 (Figure 10B).

Overlaying the feasible mixing probability surface with TEF-corrected source means showed that consumer points concentrated along the high-probability band near small-sized fishes and shrimps, and were relatively distant from zooplankton, POM, and SOM (Figure 10C). This pattern indicates that mixtures capable of reproducing the observed consumer signatures require relatively high δ^15^N and comparatively enriched δ^13^C; suspension-derived sources alone (zooplankton, POM, SOM) do not explain the isotopic signal of *M. senile*. A single 60% contour drawn on the bivariate plot (Figure 10D) encompassed most samples. Source means with standard deviation bars showed good separation among sources in the δ^13^C–δ^15^N plane, consistent with the probability surface in Figure 10C. Overall, the mixing-space diagnostics support two main source categories for *M. senile*: small-bodied and juvenile teleosts and invertebrates and suspension-derived sources, with small-sized fishes and shrimps essential to explain its elevated trophic position.

### 3.5. Bayesian Mixing Model

**Figure 11:**
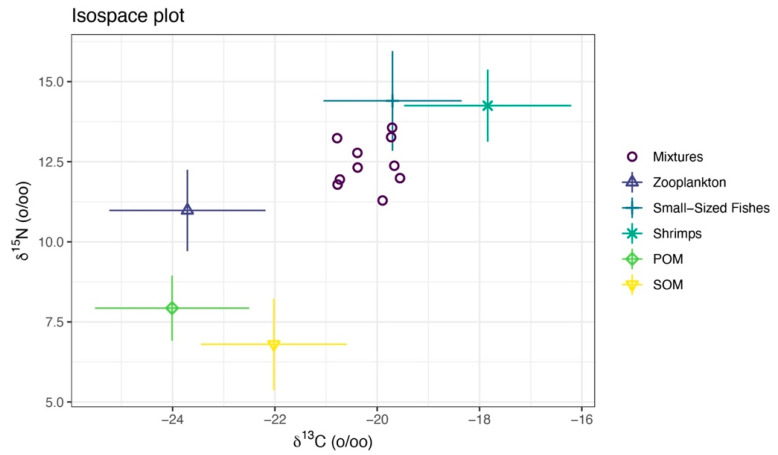
Isotopic mixing space (δ^13^C-δ^15^N) for the consumer and TEF-corrected sources. The points labeled “Mixtures” are sea anemones (*M. senile*). Symbols with error bars denote TEF-corrected source means with standard deviations. The convex hull of the corrected sources defines the feasible mixing polygon and shows whether the consumer falls within this polygon and which sources are isotopically proximate.

**Figure 12:**
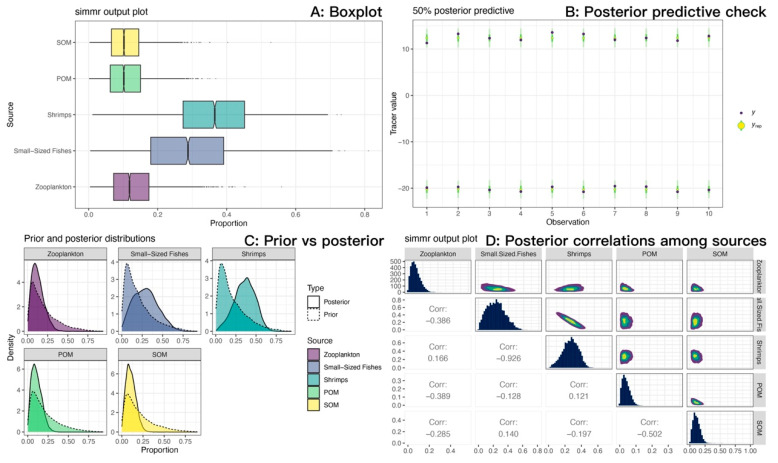
Source contribution estimates for sea anemones (*M. senile*) from the Bayesian stable isotope mixing model (SIMMR) in the North Yellow Sea. (**A**) Boxplots of posterior source contributions (central tendency and uncertainty). (**B**) Posterior predictive check: observed tracers (y) versus posterior predictive replicates (y_rep); close agreement indicates adequate model fit. (**C**) Prior versus posterior: posteriors diverge from near-uniform priors, indicating data-driven inference rather than prior influence. (**D**) Posterior correlations among sources (pairs plot): a strong negative correlation between small-sized fishes and shrimps reflects limited isotopic separability; their combined fishery-organism contribution is more stable than either source alone.

We measured the carbon and nitrogen stable isotope characteristics of a total of 84 samples (Table 7). The isotopic mixing space (δ^13^C-δ^15^N) for the consumer and TEF-corrected sources is shown in Figure 11. For *M. senile*, the Bayesian mixing model estimated that long-term nutrition was dominated by fishery-organism sources, with shrimps plus small-sized fishes contributing a combined posterior mean of approximately 0.65; mean contributions from zooplankton, POM, and SOM were 0.13, 0.11, and 0.11, respectively (Figure 12A; Table 8). Posterior predictive checks closely matched the observed δ^13^C and δ^15^N values, indicating good model fit (Figure 12B). The data volume was adequate and inference was not prior-driven; posterior densities for source contributions showed little bimodality (Figure 12C). The pairwise posterior correlation between small-sized fishes and shrimps was strongly negative (approximately −0.93), so individual contributions are uncertain, but their sum is robust (Figure 12D). Overall residual standard deviations were 0.72‰ for δ^13^C and 0.86‰ for δ^15^N, which exceed analytical error (about 0.1–0.2‰), consistent with individual heterogeneity and uncertainty in sources and TEFs. Taken together, the model results are credible.

## 4. Discussion

### 4.1. Baseline Selection and Trophic Structure

In stable-isotope studies, the choice of baseline taxa directly affects the reliability of trophic position estimates and inference on energy pathways [13,32]. Practice varies by marine system: in estuaries and embayments, year-round primary consumers or benthic fauna with stable diets are typically used, with POM as an alternative baseline [33,34,35]; on continental shelves, zooplankton or low-mobility primary consumers are commonly used [36,37]; in the open ocean, zooplankton, POM, or low-mobility primary consumers are typical [1,32,38]; and in the deep sea, where photosynthetic primary production is absent, POM is generally adopted [39,40,41]. Although no universal standard exists, there is broad consensus that suitable baselines should exhibit low mobility, relatively stable biomass, narrow and consistent diets, and ease of collection [42]. Where multiple food chains coexist, using multiple baselines helps reduce systematic bias [12,42].

In isotopic mixing space, the demersal food web of the North Yellow Sea exhibits a community-level gradient from the lower left to the upper right (Figure 4). Basal producers (phytoplankton, POM, SOM) cluster in the lower-left sector (carbon range, CR = −25.40 to −21.88‰; nitrogen range, NR = 1.53 to 7.33‰), indicating a relatively narrow spectrum of basal carbon sources. By contrast, consumers span a much wider δ^13^C range (CR = −25.15 to −15.40‰; mean ± SD = −18.88 ± 1.52‰), consistent with differentiated trophic pathways in which benthic carbon, in addition to planktonic carbon, contributes substantially. This pattern implies the presence of multiple effective baselines and supports the use of multiple baselines in trophic calculations.

Consistent with this pattern, we found that most demersal consumers in the North Yellow Sea relied heavily on small benthic gastropods and bivalves (Figure 5). These taxa co-occur with demersal consumers, show limited seasonal variability, and have relatively stable source signatures, so they are suitable baselines for the benthic pathway. By contrast, jellyfishes are pelagic; their isotopic position and resource use indicate that zooplankton is the more appropriate baseline for the pelagic pathway. Using zooplankton as a single, universal baseline would overestimate the TP of demersal consumers, whereas using gastropods and bivalves alone would underestimate the TP of jellyfishes. We therefore adopted two baselines to match the distinct trophic pathways.

Compared with earlier estimates for Yellow–Bohai fisheries species [43,44], trophic positions in our demersal assemblage are generally lower. Several non-exclusive explanations are plausible: (i) long-term declines in mean trophic level (MTL) under climate and fishing pressures; for example, global landings fell from 3.42 to 3.31 during 1950–2000 [45], regionally MTL declined by 0.16 per decade in South Australia during 1951–2010 [46], from about 4.4 to 3.8 in the East China Sea during 1974–1991 [47], and from 4.36 in 1956 to 3.8 in 2014 in the Yellow–Bohai Seas [48]; (ii) our samples are dominated by demersal taxa and include few apex predators; (iii) sampling in spring, when small-sized fishes and juveniles are common and tend to have lower TP; and (iv) methodological differences between stomach-content and stable-isotope approaches, including different integration windows and discrimination factors, which can shift TP estimates. Notably, although community TP was generally low, the trophic position of *M. senile* was 3.09 ± 0.25 (mean ± SD), higher than the value reported for anemones in the Yellow Sea (≈2.4) and consistent with recent work placing *M. senile* at trophic level 3 in the benthic food web [9,49]. Reported predators of sea anemones include sea turtles, some carnivorous sea stars, predatory gastropods, nudibranchs, crabs, and certain fishes such as butterflyfishes [50,51,52,53,54,55]. In our dataset, however, *M. senile* exceeded sea stars, gastropods and bivalves, and crabs in TP, and we did not encounter sea turtles, nudibranchs, or the fish taxa listed above. Together, these observations suggest limited top-down control on *M. senile* in the North Yellow Sea, which may help explain its biomass expansion. Sea anemones can also deter predators with stinging tentacles, and some species are capable of relocating by detaching, drifting with currents, and reattaching, further reducing predation risk [56,57,58].

### 4.2. From Passive Suspension Feeding to an Opportunistic Generalist

As carnivorous invertebrates, sea anemones consume a broad spectrum of prey (e.g., zooplankton, crustaceans, fishes) that scales with their body size [59]. Prey capture is mediated by tentacles bearing cnidocytes; upon stimulation, nematocysts deliver toxins that immobilize prey before digestion in a single gastrovascular cavity [56]. This feeding morphology requires prey to fall within an ingestible size range, tightly coupling diet to individual body size [59]. With high species diversity and a global distribution, sea anemones exhibit considerable dietary adaptability across habitats [60]. Studies on the diet of the *M. senile* are sparse. Traditionally, *M. senile* has been classified as a passive suspension feeder that relies on plankton and detritus delivered by currents [4,5,6]. Other work suggests it can take small-bodied and juvenile teleosts and invertebrates, and laboratory trials have reported shrimp consumption with light-dependent variation in predatory behavior [7,8]. Multiple lines of evidence from this study indicate a more complex and flexible trophic niche. *M. senile* occurs at trophic level 3 and occupies a relatively high position within the North Yellow Sea benthic assemblage. In isotopic space it clustered with cephalopods and medium-sized fishes, consistent with shared energy pathways and prey. Given its sessile habit and tentacle-based ambush feeding, it is reasonable to infer opportunistic carnivory: within colonized habitats (rock outcrops or anthropogenic seabed macro-debris), accessible prey within its ingestible size range may be taken.

Mixing-space diagnostics and the Bayesian mixing model further quantified this pattern. Small size-class fishery organism prey sources, specifically small-sized fishes and shrimps were essential to reproduce the elevated δ^15^N of *M. senile*. Their combined contribution had a posterior mean of approximately 0.65, whereas suspension-derived sources (zooplankton, POM, SOM) summed to about 0.35 (Figure 12A; Table 8). These estimates accord with Teng et al. (2022), who reported larger contributions from benthic shrimps followed by juvenile fishes based on isotope evidence [8]. Because of C-N mixing geometry and the proximity of the two animal sources in isotopic space, the posterior contributions of small-sized fishes and shrimps were strongly negatively correlated, but their sum was stable and provides a useful quantitative indicator for ecological interpretation and management. The mean trophic position of *M. senile* in the North Yellow Sea was higher than earlier regional values reported for anemones [49]. Part of this difference likely reflects methodology, since stomach contents capture recent ingestion whereas stable isotopes integrate assimilation over longer periods. It may also signal an adaptive dietary shift from primarily passive suspension feeding toward opportunistic predation.

### 4.3. Diet Reconstruction Under Limited Prior Information

Stable isotope analysis and Bayesian mixing models are widely used in food web studies, but they typically presume substantial prior knowledge of dietary sources [61,62]. For “prior-poor” targets such as many marine invertebrates, stomach content data often fail to provide reliable end members, and direct application of mixing models can lead to non-separable sources and results that are difficult to interpret [26,61,63]. To address this, we developed a two-stage integrative framework. First, we use trophic position analysis, isotopic niche overlap, and mixing-space diagnostics based on spatial point pattern analysis to identify functionally equivalent sets of candidate sources (qualitative screening). Second, we estimate their long-term contributions with a Bayesian mixing model (quantitative constraint). By separating “who is likely eaten” from “how much is assimilated over time,” the framework improves discriminability and interpretability when prior information is limited.

Both mixing space diagnostics and the Bayesian mixing model demonstrate that small-bodied and juvenile teleosts and invertebrates (small-sized fishes and shrimps) are essential to explain the elevated trophic position of *M. senile*. However, a strong negative correlation (Corr = −0.926) was observed between the two sources, indicating limited isotopic separability. As a result, the model trades off their estimated contributions against one another (Figure 12D). Univariate posterior distributions for both small-sized fishes and shrimps were broad, reflecting considerable uncertainty in their individual contributions, whereas their combined contribution remained stable. We therefore aggregated them into a single “small-bodied and juvenile teleosts and invertebrates” source for ecological interpretation. When sources cannot be reliably distinguished, ecological inference should focus on functionally equivalent prey groups: *M. senile* derives most of its nutrition from small-bodied and juvenile teleosts and invertebrates. This functional conclusion offers greater insight into its opportunistic generalist strategy and the resource base supporting its population expansion than would a fine-scale partition between fishes and shrimps.

To assess sensitivity to expanding the small size-class fishery organism set, we fit a six-source model that added gastropods and bivalves as an additional food source. Model fit showed no systematic improvement relative to the five-source model (deviance 47.9 vs. 48.9). The combined contribution of small-bodied and juvenile teleosts and invertebrates remained approximately 0.65 (posterior means: small-sized fishes 0.27, shrimps 0.25, gastropods and bivalves 0.16), indicating that the addition mainly diluted shares within the small-bodied and juvenile teleosts and invertebrates prey pool. The negative correlation between small-sized fishes and shrimps weakened from −0.93 to −0.69 but remained substantial; gastropods and bivalves were moderately negatively correlated with both (−0.25 to −0.44). The 95% credible interval for gastropods and bivalves was wide (0.022–0.369) and included values near zero, indicating uncertainty about the need to include this source. From a feeding perspective, adult gastropods and bivalves are not typical ingestible prey for sea anemones, so their signal likely reflects a benthic baseline rather than direct predation. We therefore excluded gastropods and bivalves from the main model and retained the six-source specification only as a sensitivity analysis. Under both specifications, the combined small-bodied and juvenile teleosts and invertebrates prey contribution was approximately 0.65, and the main conclusion was robust.

In future work, discrimination can be improved by adding other tracers. Additional isotope dimensions, such as δ^34^S or δ^2^H, or compound-specific amino acid isotope analysis (CSIA-AA) can help. In marine systems, δ^34^S is often near orthogonal to C and N and can separate pathway signals [64,65]. CSIA-AA reduces baseline drift and yields trophic position estimates, thereby narrowing source uncertainty [66,67,68,69]. A second avenue is to incorporate more informative priors on sources. Molecular diet analysis can identify the taxa that make up the small size-class fishery organism pool, supplying the Bayesian model with sharper priors and improving source attribution [70,71].

### 4.4. Ecological and Management Implications

This study indicates that the plumose anemone *M. senile* occupies a relatively high trophic position in the North Yellow Sea and relies more heavily on small-bodied and juvenile teleosts and invertebrates as food. If *M. senile* maintains sustained predation on small-sized fishes and shrimps, several effects are plausible: (i) reduced fishery recruitment, because predation on juveniles and even demersal eggs can lower early survival; (ii) top-down control on key forage taxa and resource competition with commercial fishes through consumption of important prey groups, with indirect consequences for fish yields; and (iii) restructuring of habitat and benthic community composition, as dense anemone blooms at relatively high trophic position, with little predation from higher levels, can modify the benthic environment. These inferences highlight the need to evaluate the strength of anemone–fishery interactions and their potential cascading effects in management planning.

We recommend listing the plumose anemone *M. senile* as a priority species in fishery monitoring for the North Yellow Sea. Specifically: (i) track population density and mean body length, given the close link between body size and ingestible prey size; (ii) quantify spatiotemporal co-occurrence with early life stages of fishes and with key forage taxa; (iii) use ecosystem models to assess carrying-capacity thresholds and assign ecological risk levels; (iv) because anthropogenic seabed macro-debris (ASMD) likely facilitates outbreaks, implement pilot actions for targeted anemone removals and ASMD cleanups in priority habitats such as protected areas, nursery grounds, and spawning grounds, aligned with existing policies, and strengthen regulations to reduce inputs of marine litter.

## 5. Conclusions

Under limited prior information, we developed an integrative framework that couples trophic position estimation, isotopic niche analysis, spatial point-pattern diagnostics of the mixing space and a Bayesian mixing model to reconstruct the diet of the plumose anemone *M. senile* in the North Yellow Sea. The trophic position of *M. senile* was 3.09 ± 0.25 (mean ± SD), and its isotopic niche was close to those of cephalopods and medium-sized fishes. The Bayesian model attributed most long-term nutrition to small-bodied and juvenile teleosts and invertebrates, with small-sized fishes plus shrimps contributing a combined posterior mean of approximately 0.65, while suspension-derived sources (zooplankton, POM, SOM) contributed less. These results support an opportunistic generalist rather than a strict suspension feeder. This feeding mode could weaken early fish recruitment, impose top-down control on key forage taxa, and restructure benthic habitats. We recommend monitoring *M. senile* (size structure, population density, and co-occurrence with juveniles and forage biota) and piloting targeted removals and ASMD cleanups in critical habitats. The framework is applicable to diet reconstruction when prior information is limited; incorporating δ^34^S, CSIA-AA, and molecular diet analysis evidence should further sharpen source discrimination.

## Figures and Tables

**Table 1:** Functional groups and representative taxa of the demersal food web in the North Yellow Sea.

Label	Functional Group	Species Composition
**a**	**Phytoplankton**	Phytoplankton
**b**	**Zooplankton**	Zooplankton
**c**	**POM**	Suspended particulate organic matter
**d**	**SOM**	Sedimentary particulate organic matter
**e**	**Jellyfishes**	Moon jelly (*Aurelia aurita*), Flame jellyfish (*Rhopilema esculentum*), etc.
**f**	**Brittle Stars**	Notched brittle star (*Ophiura sarsii*)
**g**	**Sea Stars**	North Pacific seastar (*Asterias amurensis*) and *Luidia yesoensis*
**h**	**Sea Urchins**	Hardwick’s sea urchin (*Temnopleurus hardwickii*), Cardiac sea potato (*Echinocardium cordatum*), and Striped spine sea urchin (*Temnopleurus toreumaticus*)
**i**	**Sea Anemones**	*Metridium senile*
**j**	**Gastropods** **and B** **ivalves**	Large weathervane scallop (*Mizuhopecten yessoensis*), *Volutharpa perryi*, Purple whelk (*Rapana venosa*), and Bladder moon snail (*Glossaulax didyma*)
**k**	**Cephalopods**	Japanese squid (*Loliolus japonica*), Butterfly bobtail (*Sepiola birostrata*), Mimika bobtail squid (*Euprymna morsei*), Golden cuttlefish (*Sepia esculenta*), Whiparm octopus (*Octopus variabilis*), and Gold-spot octopus (*Amphioctopus fangsiao*)
**l**	**Shrimps**	Hakodate sand shrimp (*Crangon affinis*), *Eualus sinensis*, Kishi velvet shrimp (*Metapenaeopsis dalei*), Chinese ditch prawn (*Palaemon gravieri*), Southern rough shrimp (*Trachypenaeus curvirostris*), Japanese snapping shrimp (*Alpheus japonicus*), Lesser glass shrimp (*Leptochela gracilis*), Isada krill (*Euphausia pacifica*), etc.
**m**	**Mantis Shrimps**	Japanese squillid mantis shrimp (*Oratosquilla oratoria*)
**n**	**Crabs**	Edward’s hermit crab (*Diogenes edwardsii*), Graceful decorator crab (*Oregonia gracilis*), Gibbous rock crab (*Romaleon gibbosulum*), Two-spot swimming crab (*Charybdis bimaculata*), etc.
**o**	**Small-Sized Fishes**	Japanese anchovy (*Engraulis japonicus*), Scaly hairfin anchovy (*Setipinna taty*), Pacific sandlance (*Ammodytes personatus*), Yellow croaker (*Larimichthys polyactis*), Pygmy sculpin (*Cottus paulus*), Smallhead hairtail (*Eupleurogrammus muticus*), Ocellate spot skate (*Okamejei kenojei*), Indian perch (*Jaydia lineata*), Japanese sillago (*Sillago japonica*), Whitespotted dragonet (*Callionymus beniteguri*), *Pholis fangi*, *Amblychaeturichthys hexanema*, etc.
**p**	**Medium-Sized Fishes**	Tanaka’s snailfish (*Liparis tanakae*), Silver croaker (*Pennahia argentata*), Whitespotted conger (*Conger myriaster)*, Pacific cod (*Gadus macrocephalus*), Sôhachi (*Cleisthenes pinetorum*), Yellow goosefish (*Lophius litulon*), Eelpout (*Zoarces viviparus*), Korean rockfish (*Sebastes schlegelii*), Fat greenling (*Hexagrammos otakii*), Silver pomfret (*Pampus argenteus*), Japanese seabass (*Lateolabrax japonicus*), Bartail flathead (*Platycephalus indicus*), Ridged-eye flounder (*Pleuronichthys cornutus*), Bluefin gurnard (*Chelidonichthys kumu*), Whitespotted conger (*Conger myriaster*), *Hemitripterus villosus*, *Chirolophis japonicus*, Yellowfin pufferfish (*Takifugu xanthopterus*), Spotted halibut (*Verasper variegatus*), etc.

**Table 2:** Trophic position of functional groups in the demersal food web of the North Yellow Sea.

Label	Functional Groups	Sample Size (n)	Mean of TP	SD of TP	TL
a	Phytoplankton	6	1.34	0.16	1
b	Zooplankton	5	1.97	0.20	2
c	POM	4	1.08	0.07	1
d	SOM	10	0.63	0.29	1
e	Jellyfishes	3	2.51	0.10	3
f	Brittle Stars	4	1.78	0.34	2
g	Sea Stars	6	2.36	0.20	2
h	Sea Urchins	7	1.80	0.43	2
i	Sea Anemones	10	3.09	0.25	3
j	Gastropods and Bivalves	13	2.05	0.40	2
k	Cephalopods	21	2.93	0.24	3
l	Shrimps	18	2.51	0.14	3
m	Mantis shrimps	10	3.23	0.18	3
n	Crabs	15	2.80	0.22	3
o	Small-Sized Fishes	36	2.71	0.32	3
p	Medium-Sized Fishes	70	3.02	0.27	3

**Table 3:** Posterior probabilities that an individual from the row group falls within the isotopic niche of the column group (*α* = 95%).

	Sea Anemones	Small-Sized Fishes	Medium-Sized Fishes
Sea Anemones	NA	52.68	63.04
Small-Sized Fishes	21.57	NA	78.31
Medium-Sized Fishes	24.08	85.81	NA

**Table 4:** Posterior probabilities that an individual from the row group falls within the isotopic niche of the column group (α = 95%).

	Sea Anemones	Gastropods and Bivalves	Cephalopods
Sea Anemones	NA	0.07	78.30
Gastropods and Bivalves	0.02	NA	18.13
Cephalopods	19.26	16.36	NA

**Table 5:** Posterior probabilities that an individual from the row group falls within the isotopic niche of the column group (α = 95%).

	Sea Anemones	Shrimps	Mantis Shrimps	Crabs
Sea Anemones	NA	1.76	2.66	13.87
Shrimps	2.87	NA	0.84	68.22
Mantis shrimps	3.16	0.74	NA	59.37
Crabs	8.87	37.77	29.67	NA

**Table 6:** Posterior probabilities that an individual from the row group falls within the isotopic niche of the column group (α = 95%).

	Sea Anemones	Jellyfishes	Brittle Stars	Sea Stars	Sea Urchins
Sea Anemones	NA	0.00	1.32	3.86	1.97
Jellyfishes	0.01	NA	0.01	14.16	75.64
Brittle Stars	0.24	0.00	NA	1.45	44.06
Sea Stars	3.36	1.29	2.63	NA	55.89
Sea Urchins	0.23	1.42	6.81	18.33	NA

**Table 7:** Values of δ^13^C and δ^15^N for the potential food sources and consumers.

Category	Number	Mean of δ^13^C	SD of δ^13^C	Mean of δ^15^N	SD of δ^15^N
Small-Sized Fishes	36	−20.10‰	0.36‰	11.00‰	1.19‰
Shrimps	18	−18.24‰	0.99‰	10.58‰	0.52‰
Zooplankton	5	−24.11‰	0.80‰	7.58‰	0.78‰
POM	5	−24.41‰	0.76‰	4.53‰	0.20‰
SOM	10	−22.42‰	0.59‰	3.40‰	1.03‰
Sea Anemones	10	−20.16‰	0.50‰	12.45‰	0.74‰

**Table 8:** The analysis results of Simmr.

Food Source Category	Mean ± SD (‰)	95% Credible Interval (‰)	R-Hat Values
Small-Sized Fishes	29.7 ± 14.8	4.4–61.1	1
Shrimps	35.6 ± 12.7	9.0–58.7	1
Zooplankton	13.2 ± 7.7	2.1–30.9	1
POM	10.7 ± 6.0	1.8–24.2	1
SOM	10.8 ± 5.7	2.0–23.5	1

## Data Availability

The species data collected and analyzed during this study are available in this paper. The original data may be supplied by the corresponding author upon request.
